# Planning of a Health Emergency Disaster Risk Management Programme for a Chinese Ethnic Minority Community

**DOI:** 10.3390/ijerph16061046

**Published:** 2019-03-22

**Authors:** Greta Tam, Emily Ying Yang Chan, Sida Liu

**Affiliations:** 1 Jockey Club School of Public Health and Primary Care, The Chinese University of Hong Kong, Hong Kong, China; gretatam@cuhk.edu.hk; 2 Collaborating Centre for Oxford University and CUHK for Disaster and Medical Humanitarian Response (CCOUC), The Jockey Club School of Public Health and Primary Care, The Chinese University of Hong Kong, Hong Kong, China; liusida2008@gmail.com; 3 Nuffield Department of Medicine, University of Oxford, Oxford OX3 7BN, UK; 4 FXB Center of Health and Human Rights, Harvard University, Boston, MA 02115, USA

**Keywords:** ethnic minority, China, Health-EDRM

## Abstract

Rural populations living in poverty are the most vulnerable to disaster. Despite this increased risk of recurrent disaster, previous disaster experience is not a good predictor for disaster preparedness in these populations. This was evidenced on 31 August 2012, when a major flood occurred in Sichuan, China. A health needs assessment carried out in December 2012 showed that residents of Hongyan village, a Yi-minority community in Sichuan lacked disaster preparedness. This indicated that measures were necessary to improve Health Emergency Disaster Risk Management (Health-EDRM) in the community. Nutbeam’s planning model for health promotion was used to guide the development of a Health-EDRM programme at Hongyan Village, Liangshan Yi Autonomous Prefecture, Sichuan. Relevant information was obtained from sources such as literature review, household surveys and stakeholder interviews. A team of stakeholders conducted an interactive workshop to train villagers on disaster preparedness in March 2014. Disaster kits and equipment for Oral Rehydration Solution preparation were handed out to villagers.

## 1. Introduction

### 1.1. Disaster Health Preparedness in the Rural Poor Areas in China

Globally, 75% of people living below the poverty line of US $1.07/day live in rural areas. Despite economic growth in developing countries, the resulting benefits are not spread evenly. Poverty is becoming increasingly ruralized in China, Eastern Europe and Central Asia. Rural populations living in poverty are the most vulnerable to disaster. Climate change increasingly exposes rural areas to weather-related shocks and stresses (e.g., drought and repeated flooding) [1]. Meanwhile, poverty causes decreased disaster resilience due to lack of access to services (e.g., health care and education) and infrastructure (e.g., water and sanitation) [2]. Despite the increased risk of recurrent disaster among the rural poor, previous disaster experience is not a good predictor for disaster preparedness in these populations [3,4,5].

### 1.2. Building Disaster Health Resilient Communities

In 2015, the UN General Assembly endorsed the Sendai Framework for Disaster Risk Reduction. Priorities for Action included understanding disaster risk and enhancing disaster preparedness [6]. This built upon the previous Hyogo Framework for Action (2005–2015), which included as one of its priorities “use knowledge, innovation and education to build a culture of safety and resilience at all levels” [7].

Resilience is “the ability of a system, community or society exposed to hazards to resist, absorb, accommodate, adapt to, transform and recover from the effects of a hazard in a timely and efficient manner, including through the preservation and restoration of its essential basic structures and functions through risk management” [8]. The United Nations and Red Cross have advocated community-based disaster preparedness programmes to build disaster resilient communities [9,10]. This approach combines integrated programming and cooperation with local communities. Integrated programming ensures a holistic approach: elements from different sectors (e.g., health and hygiene education and disaster preparedness) are combined into one programme. Consulting local villagers ensures that interventions are tailored to specific community needs. Although efforts have been made to implement Health-EDRM programmes globally [9,10,11], Asia is still disproportionately affected by disasters: China, Indonesia, Philippines and India are among the top five countries most prone to natural disasters and Asia accounts for 90.1% of disaster victims [12].

### 1.3. Local Epidemiological and Demographic Data of Hongyan Village, Liangshan Yi Autonomous Prefecture, China

In the last decade, China has experienced the most natural disasters in the world [12]. Under the effects of climate change, there has been an increase of weather-related disasters by 69% globally in the last decade, with floods becoming increasingly frequent in China. Many remote villages in China have limited health and hygiene awareness and disaster preparedness, due to the low education level and lack of information. In addition, many lack basic sanitation infrastructures, such as proper latrines and access to basic medical care. Consequently, there is a risk of poor sanitation after a flood, as surface and groundwater are contaminated by effluent from latrines [10]. 

**Figure 1:**
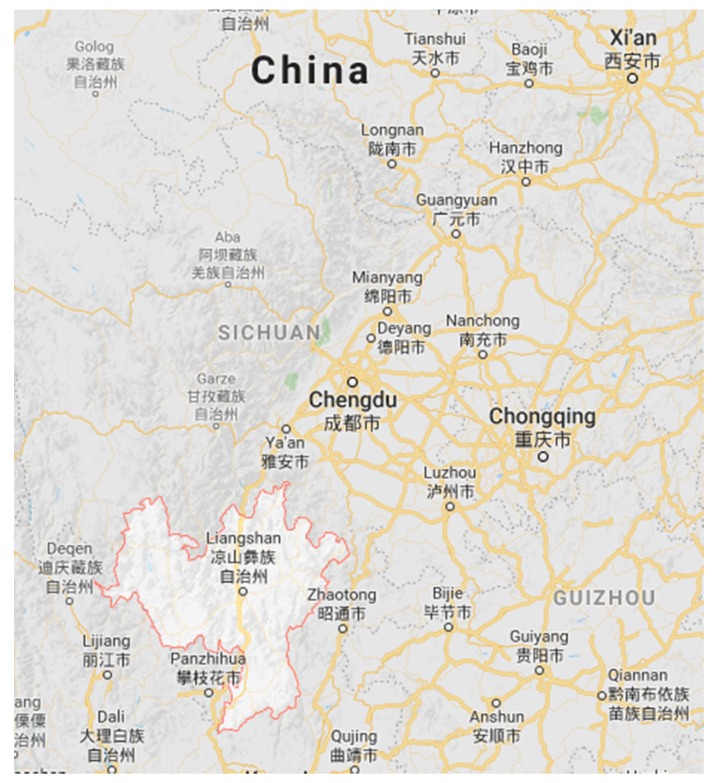
Geographical location of Sichuan Province and Liangshan Yi Autonomous Prefecture.

Hongyan village is one of 169 villages in Xide county, under the jurisdiction of Liangshan Yi Autonomous Prefecture, in the southern area of Sichuan province. Hongyan village is 5 km from the closest township, Lianghekou. Figure 1 shows a location map for the case area. It is composed of 4 sub-groups, with 218 households and 826 residents. The villagers live on the bank of Sunshui River, in a mountainous landscape with poor road conditions. Liangshan has the largest community of Yi ethnic minority group in China. Yi ethnic groups live mostly in rural and mountainous areas in Sichuan, Yunnan, Guizhou and Guangxi. Education levels are low in Yi ethnic groups and they speak their own language [13,14].

A major flood occurred on 31st August 2012 at Xide County, causing great damage: 218,000 residents were affected, 13,300 houses collapsed, and 29,000 houses were seriously damaged, while 1 death and 2 missing people were reported. Flooding also damaged the infrastructure, including roads, water supply, telephone and broadcasting. The county was therefore temporarily isolated from external information and assistance. In 2012, the Collaboration Centre for Oxford University and CUHK for Disaster and Medical Humanitarian Response (CCOUC) was invited by Wu Zhi Qiao Charitable Foundation to perform a health needs assessment and health intervention in Hongyan village.

This study aimed to identify and use relevant data to plan a Health-EDRM programme for Yi-minority community in Sichuan Province, China.

## 2. Materials and Methods 

### 2.1. Planning Framework and Data Collection

**Figure 2:**
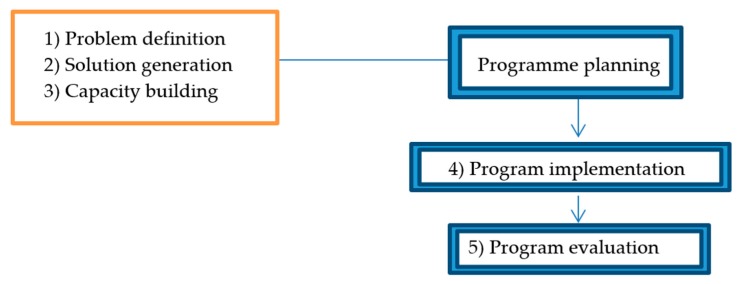
Three planning steps within Nutbeam’s model for health promotion.

Nutbeam’s model for health promotion was employed for planning the health promotion programme (Figure 2). The model was created with the intention to help systematically link relevant research and theory to the practicalities of programme implementation and evaluation. The model was separated into three parts: (i) problem definition; (ii) solution generation; and (iii) capacity building. Problem definition aims to clarify what and who are the targets of the health-EDRM programme. Solution generation aims to determine how and when change could be achieved in the target population. Capacity building aims to create the best conditions for the health-EDRM programme through the assessment of resources (such as financial and human resources) to ensure the programme objectives are a good fit for the available resources. These 3 planning stages require unique information and are summarized in Table 1.

### 2.2. Information for Health Planning

#### 2.2.1. Literature Review

The literature review identified epidemiological data and relevant past studies. This aided description of the local situation and people, their behaviour, and any predictors for lack of disaster preparedness. In addition, this provided evidence for choosing the most appropriate theory and intervention model to improve Health-EDRM. MEDLINE, Embase and Google were searched for academic and grey literature, and titles were screened for relevance. To review the literature on local epidemiological and demographic data, the keywords “China” and “disaster” were used. Articles and websites were limited to those published between 2011 and 2016. To review the literature on the determinants of disaster preparedness, the keywords “severity”, “diarrhoea”, “treatment” and “household disaster preparedness” were used. To review the literature for the solution generation stage, the keywords “theory”, “disaster preparedness” and “disaster risk communication” were used. Articles were limited to systematic reviews.

#### 2.2.2. Household Survey

With limited information published about health status of people in the Liang Shan area, CCOUC conducted a field-based health needs assessment in Hongyan village of Xide county, Liangshan, Sichuan in December 2012. Cross-sectional household surveys were carried out to assess health status, health service availability and utilization of healthcare. A follow-up trip was conducted in March 2014. Cross-sectional household surveys were administered, covering demographics, health and access to health care, as well as knowledge, attitudes and practices of Health-EDRM. The surveys were administered face-to-face. For the 2012 survey, households were recruited using snowball sampling, and the last birthday method was used to recruit a participant within the household. The resulting sample size was 54. 52% were male and 48% female. The mean age of respondents was 43.6 years, with a maximum of 77 years and a minimum of 18 years. All were people of Yi ethnicity except one, who identified as a person of Miao ethnicity. 98% were local farmers and 2% worked as labour workers. 67.9% were illiterate, 11.3% received no formal education while 13.2% and 7.6% received primary and secondary education respectively. For the 2014 survey, all participants of the health-EDRM programme were recruited. The resulting sample size was 100. The respondent profile is reported in another paper [15].

#### 2.2.3. Focus Group

One female and one male focus group (each consisting of 6–8 villagers) were studied. Participants were recruited using snowball sampling. Participants were asked what they would do if they felt sick, any barriers they encountered towards seeking healthcare, how they prepared for disasters, and what their response was during the previous flood. Focus groups were semi-structured. Ethics approval was obtained from the Joint Chinese University of Hong Kong—New Territories East Cluster Clinical Research Ethics Committee (ref no. 2016.334).

#### 2.2.4. Discussion with Stakeholders

The sectors and stakeholders involved, and their roles and expertise, are summarized in Table 2.

A participatory research approach was used. The only external stakeholders were from the Architecture/housing sector: WZQ and the Department of Architecture, CUHK. The study team was composed of CCOUC staff and students from CUHK. Data analysis was also conducted by the study team.

### 2.3. Data Analysis

Articles and websites were screened for relevance to the information needed for the literature review. Data synthesis was by exploration of the application of the data to the planned health-EDRM programme in a narrative summary. Survey data were double entered and cleaned by trained staff. Descriptive statistics were generated using SPSS version 21.0. Focus group discussions were taped and transcribed into verbatim. A member of the health needs assessment team and the first author (G.T.) reviewed the transcript and carried out thematic analysis independently. G.T. compared the two sets of thematic analyses. Since the analysis carried out by the team member was for the purpose of writing a trip report, while the author’s purpose was to summarize research according to the research framework, the author selected the final themes that were relevant to this study. 

Data from the different sources were integrated according to common themes for each category of information needed into narrative summaries.

## 3. Results

### 3.1. Problem Definition: Community Needs and Perceived Priorities

Table 3 presents the community needs and perceived priorities in Hongyan village. The results suggest that gastrointestinal problems are common, especially during flood. Poverty and lack of infrastructure result in inadequate health care access, disaster prevention and response systems. Villagers lack empowerment to protect their family’s health and safety during a disaster.

### 3.2. Problem Definition: Determinants of Lack of Disaster Preparedness

Literature review and survey of villagers provided information on patterns of gaps in lack of disaster preparedness in Hongyan village, which were used to design a tailored Health-EDRM programme. The following themes were identified:

#### 3.2.1. Theme 1: Knowledge of Consequences and Treatment of Diarrhoea

Yoder et al. reported that in the home setting in developing countries, if diarrhoea was perceived as serious, treatment was more likely to be given [16]. In our survey, although most knew severe diarrhoea resulted in dehydration, fewer respondents realized the potential extent of severity. Only 17.6% of respondents had heard of Oral Rehydration Solution (ORS). This suggests Hongyan villagers lack knowledge regarding the potential severity of diarrhoea and its treatment. The further lack of knowledge of how to make ORS suggests that administering ORS is rarely put into practice in Hongyan village.

#### 3.2.2. Theme 2: Knowledge, Attitude and Practice towards Use of Disaster Kit

CDC reported that household disaster preparedness (such as owning a disaster kit) was associated with disaster preparedness knowledge and attitudes [17]. In our survey, although 53.7% thought it necessary to prepare a disaster kit and 42.6% would consider preparing a disaster kit, only 24% already had a disaster kit and 16.7% knew how to prepare a disaster kit. This suggests that villagers were willing to prepare a disaster kit, but were hindered by lack of knowledge. Even if they possessed a disaster kit, the contents may be inadequate.

### 3.3. Solution Generation

#### 3.3.1. Choice of Health Promotion Model and Intervention Strategy

A systematic review of the application of behavioural theories to health-EDRM reported that the Health Belief Model (HBM) was one of the most commonly applied models [18]. Under this model, five beliefs are required for an individual to exhibit behaviour change:

1. That they are susceptible to the problem.
2. That the problem could result in potentially severe consequences.
3. That course of action can reduce risks.
4. That benefits of action outweigh barriers
5. Self-efficacy [19].

Studies have shown that perception of susceptibility, severity, benefits and barriers influence predicted preparedness for disease outbreaks [20,21,22] and disaster (e.g., possessing a disaster kit) [23]. In planning this health promotion programme, the content followed the Health Belief Model constructs.

A systematic review of intervention studies of disaster risk communication reported that interventions with community involvement improved disaster preparedness [24]. This included community participation approach (training villagers in disaster preparedness) [25] and small group discussions with health promoters [26]. In addition, interventions using games led to increased knowledge of disasters [27,28,29]. In this programme, we planned to include villagers in an interactive intervention using a combination of posters, hands-on demonstration and games.

#### 3.3.2. Experience from Past Programmes and Practitioners Applied to the Intervention

Since February 2009, CCOUC has been conducting health needs assessments and health interventions in disaster-prone rural poor villages in China. Based on past experience, it was decided a half-day interactive workshops would be useful to be conducted in the village. 

The workshop was conducted by eight students: Four conducted the ORS intervention and four conducted the disaster kit intervention. Teams were comprised of medical and public health students, enabling sharing different skills and knowledge. 100 villagers participated. The village chief was consulted regarding the best time and location, as we wanted to include as many villagers as possible, for the greatest impact. The location’s capacity was fully utilized, as it could accommodate 100 people. Since villagers spoke the local language, translators were recruited to facilitate communication.

The purpose of this workshop was to impart knowledge of the importance of ORS and disaster kits, as well as providing a hands-on demonstration to provide the necessary tools for self-empowerment: In the event of a disaster with poor access to external aid, villagers would still be able to take preliminary measures to improve their situation. 

The ORS intervention consisted of using a poster to illustrate the consequences of gastroenteritis and components of ORS. Villagers were invited to demonstrate ORS preparation methods. In the end, standard teaspoons and 150 mL cups were given as souvenirs to solve the problem of differing spoons and cups in households. The disaster intervention used a poster to illustrate the importance and contents of a disaster kit. Visitors played a game of identifying disaster kit contents. In the end, disaster kits were given out, and Polaroid photos were taken of family members, to aid identification during disasters. 

### 3.4. Capacity Building

#### 3.4.1. Mobilizing Human, Material and Financial Resources

Our team consisted of CCOUC staff (doctors, nurses and public health practitioners) and students (with medical and public health background). CCOUC previously conducted health needs assessment and interventions in Sichuan, Gansu and Guangxi villages. CCOUC staffs were, therefore, experienced health educators. They also organized the logistics of the trip (accommodation, meals and transport). The students had mixed experience backgrounds; some had attended previous trips while others had not. Meetings involving all team members were held before the trip to evaluate poster designs and intervention plans. In this way, more experienced team members trained those who were new. Students designed and printed posters and flyers. CCOUC staff prepared ORS souvenirs (teaspoons and plastic cups), disaster kit (lighter, penknife, medication box, thermal blanket, whistle, torch and bag), and Polaroid cameras.

The intervention was held in an open space outside the village chief’s house, as this was the biggest space available and convenient for villagers. Table 4 shows a typical example of financial resources required for the health-EDRM programme. The estimated budget in 2014 had a slight cost inflation compared to 2012, in order to take into account possible cost inflation over the years 

Expenses were solicited from various sources, including CUHK’s ICARE programme and CCOUC development fund. Although the cost of intervention per participant was relatively high due to travel expenses, the project also served other purposes: training and education of students, academic exchange, research and raising public awareness.

#### 3.4.2. Training People and Building Sustainable Programmes

Our programme provides villagers with education and equipment for disaster preparedness. Since most villagers live in poor rural areas, giving them disaster kits, ORS recipes and utensils reduces problems related to affordability and access. The equipment can be re-used, and they can demonstrate their acquired knowledge to family and friends back home. In this way, the programme trains villagers to pass on their knowledge and use their new skills in times of need.

As well as training villagers, the programme trains students and staff. CCOUC has ongoing health promotion programmes in a wide range of disaster-prone rural villages in China. This trip was part of CCOUC’s Ethnic Health Minority Project, ongoing since 2009. Due to the mix of team member experience in CCOUC trips, students and staff learn from each other. Experienced team members will take up additional responsibility in future trips. This includes leading teams and dealing with logistics. Lessons learnt from each trip inform the next trip. Graduate students could obtain academic credit for participating or as part of a global health elective “field action lab”. Some used the collected data to write their master’s thesis. Medical students were also recruited from CUHK’s Global-Physician Leadership Stream and Medical Outreachers, which aims to train holistic future physicians. Thus, this trip is a building block in multiple programmes.

#### 3.4.3. Raising Public and Political Awareness

After the flood in August 2012, WZQ and CCOUC contacted the chief of Hongyan village and visited the village on an exploration mission. The village chief agreed there was a need for health needs assessment and programme development. CCOUC staff discussed future trip logistics with him and confirmed his support, serving as the access point to Hongyan village. Public awareness of the intervention was crucial for sufficient participation. The chief helped spread the details to the villagers. Households previously approached for surveys were also informed. When the intervention was about to start, the loudspeaker microphone reminded villagers to attend.

## 4. Discussion

Lack of disaster preparedness is a problem found in many disaster-prone areas globally. Therefore, international information about disaster kits and treatment of gastroenteritis is available. However, although rural ethnic minority groups comprise a significant proportion of those living in disaster-prone areas in China, there is a lack of research focusing on these communities [30]. Literature review alerted us of the need to avoid generalizing other research findings, as there is a difference between ethnic and non-ethnic minorities: The higher illiteracy rate, different language and occupations among the Yi minority [31] meant that different strategies were needed to overcome communication barriers. Our study revealed that a worrying proportion described deteriorating general health over the years. Villagers had a poor health baseline, even in normal times outside the disaster period. Since the average age was only 42.2 and the predominant occupation was agriculture, this deterioration might affect not only their quality of life but also livelihood. Further data shed light on possible reasons for the predominantly gastrointestinal problems in the village: A similarly high proportion of respondents to those reporting diarrhoea in the last three months did not have a regular handwashing habit, despite having stable access to water sources [31]. In addition, more than half of the respondents drank water without treatment [15]. The lack of healthcare access is typical of low-income rural villages in remote areas [32,33]. Together with our findings in the determinants of lack of disaster preparedness section indicating a lack of knowledge regarding the consequences and treatment of diarrhoea, we conclude that there is a great health need in this aspect; empowering villagers through education on the severity of diarrhoea and how to make ORS could vastly improve lives by preventing dehydration, despite the lack of healthcare access. 

The low number of fatalities were likely due to the slow-onset nature of the disaster, which allowed time for evacuation. Most villagers had no preparation before the flooding and less than half felt empowered. Nevertheless, it is encouraging that the community was close-knit enough to communicate and look out for each other as a village. This could have implications for health-EDRM, as knowledge gained by the 100 villagers from the intervention could potentially be passed onto other villagers through informal conversation and advice. Most villagers were forced to move after the flood, and thus any health-EDRM items must be essentials that are easy to prepare and carry. Despite a willingness to prepare disaster kits, there was a lack of knowledge regarding preparation, thereby leading to lack of possession of disaster kits. Research shows that disaster kits may be the most cost-effective means of reducing mortality in these settings [34]. Incorporation of ORS and disaster kit preparation in a health-EDRM programme would therefore address health needs for this community. 

Apart from the Health Belief Model, another model that has been used in health-EDRM programmes is the Precaution Adoption Process Model (PAPM). One study explored the correlation between the HBM and the PAPM and found that perceived benefits and perceived barriers in the HBM predicted adoption stage in PAPM [35]. Given the emphasis on perceived barriers, future health-EDRM programmes could explore whether there are significant barriers apart from knowledge and poverty. The importance of community involvement was echoed by the World Health Organization, which listed it as a strategy for achieving the Sendai Framework priorities [36]. 

Although the project managed to gather information specific to local villagers, there are a few limitations. Our surveys had high response rates (ranging from 93–100%), but the quality of responses was hampered by language barriers. Translators were needed for surveys as most villagers could not speak Putonghua. In addition, convenience sampling was used, as households were far apart. Without random sampling, selection bias is likely. Our focus groups gathered more in-depth information regarding villagers’ opinions. However, focus groups are limited by small sample size, and villagers might concur with each other as a result of peer pressure. Our semi-structured interview with the village chief informed us of the village disaster plan and response system. However, this only represented one person’s opinion, and it was difficult to ascertain whether participant recall bias, such as inaccurate recall of events, existed. 

Although community involvement was crucial, gaining access was not easy. Securing political support from the village chief was crucial, with trust built up slowly through a previous trip and ongoing communication. The village chief was helpful in providing an overview of the village situation, local input, and village access. Planning the health promotion programme required much logistical support, using limited time and resources. Due to these pressures, team members found themselves under stress in a new environment. Pairing team members with different levels of experience and capabilities eased the adjustment of new team members. In addition, incorporating evaluation during the planning stage ensured that the programme could be thoroughly and efficiently evaluated by comparing changes before and after the program.

Health promotion during the post-disaster period was a good opportunity as villagers would be more aware of the impact of a disaster and eager to learn about disaster preparedness. However, communication was important to manage expectations. As disaster was fresh in their minds, they desired infrastructure reconstruction, which could not be delivered through this program. Nevertheless, the health promotion programme on Disaster Preparedness for Yi-minority community in Sichuan Province in China was successfully delivered in March 2014.

## 5. Conclusions

Based on this experience of using Nutbeam’s planning model to develop a tailored health-EDRM programme, future programmes could be planned and expanded to different disaster-prone areas. To be successful, it is essential that health-EDRM programmes address local health needs and priorities. Using a planning model ensures a systematic approach to planning and is useful for defining the scope of an intervention, particularly where scant pre-existing information is available regarding the local situation. The planning model can serve as a framework for organizing and combining multiple sources of information, thereby translating evidence-based planning into a health promotion programme applicable to the local context.

## Figures and Tables

**Table 1:** Summary of information needed and sources for each planning stage.

Planning Stage	Information Needed	Source	Section
Problem definition	Local epidemiological and demographic data to determine the size and nature of the problem	Literature review	1.3
	Community needs and perceived priorities	Household survey Focus group	3.1
	Determinants of lack of disaster preparedness	Literature review Household survey	3.2
Solution generation	Theories and intervention models	Literature review	3.3
	Evidence from past programmes and practitioners	Discussion with stakeholders	3.3
Capacity building	Mobilizing resources, training and infrastructure development, raising public and political awareness	Discussion with stakeholders	3.4

**Table 2:** Stakeholders and their roles.

Sector	Stakeholders	Roles	Expertise
Public health, medicine	Collaborating Centre for Oxford and CUHK for Disaster and Medical Humanitarian Response (CCOUC)	-Recruit volunteers -Co-ordinate stakeholders -Carry out the health needs assessment -Plan, implement and evaluate the program	-Multi-disciplinary (members include doctors and public health professionals) -Health needs assessment -Planning, implementing and evaluating program
Architecture/housing	Wu Zhi Qiao (WZQ) Foundation, Department of Architecture, Chinese University of Hong Kong (CUHK)	-Assessing the need for a sustainable development project (e.g., building bridges, schools and housing)	-Access to the community, local knowledge (WZQ previously conducted exploration mission on 20–22 October 2012.
Local stakeholders	Local village representatives ^1^	-Liaise with other stakeholders on behalf of local villagers -Facilitate programme planning, implementation and evaluation	-Access to the community, local knowledge
Programme volunteers	Students from CUHK	-Human resources	-Manpower

^1^ A semi-structured interview was conducted with the village head regarding disaster preparedness. His opinion was also sought regarding the feasibility of proposed interventions.

**Table 3:** The community needs and perceived priorities in Hongyan village.

Theme	Results	Source of Information ^#^
Health needs	General health: -53.7% had good health status, but 43.5% complained of deteriorating health compared to 5 years ago. Diseases requiring long-term medication: -Gastrointestinal symptoms were most frequently reported (16.7%), followed by arthritis (6.2%) and respiratory complaints (2%) -38.9% reported experiencing diarrhoea in the last 3 months	-Survey
Healthcare access	-No village doctor was available in Hongyan village. No local emergency service is available: The closest ambulance station is in Xide county, with a response time of 1 h. -For health visits, 51.8% went to the township clinic, 20.4% went to the hospital, while 3.7% preferred to buy over-the-counter medicine -Many villagers only seek medical consultation when they cannot withstand discomfort, due to the cost of medical care. -50% had avoided medical care in the past 3 months as they were unable to afford it.	-Focus group -Survey -Focus group -Survey
Health needs during a disaster	-Only 38.9% of villagers thought they had the ability to protect their family’s health and safety during a disaster -In the 2012 flood, 31.5% reported falling sick. Of those, the most common complaint was gastrointestinal symptoms (37.5%).	-Survey
Disaster preparation and response	-To prepare for disasters, regular exercises were held to demonstrate the route of evacuation. No other disaster preparation was done, due to lack of knowledge and financial support. -68.5% of respondents had no preparation before flooding. -The only warning system used mobile phones, which did not work during disasters, due to serious damage to communication infrastructure. During the 2012 flood, most villagers moved higher up the mountain to avoid landslides and house collapse. They stayed in temporary shelters for an average of 62 days.	-Focus group -Survey -Focus group

^#^ Household survey and focus groups conducted in 2012.

**Table 4:** Example of typical items and equipment to conduct field based activities in China (as of 2012).

Category	Item	Cost (USD) in 2012	Estimated Budget (USD) for 2014
Manpower for background survey and focus group (2012 trip)	Air-tickets and road transportation costs for 10 team members	7987	
	Accommodation for 10 team members and drivers	459	
	Meal expenses for 10 team members	764	
	Incentives for 54 interview participants	103	
	Honorarium for local staff	267	
Manpower for background survey and intervention (2014 trip)	Air-tickets and road transportation costs for 24 team members		19,200
	Accommodation for 24 team members and drivers		1100
	Meal expenses for 24 team members		2000
	Incentives for 100 interview participants		200
	Honorarium for local staff		300
Intervention materials	Printing 5 posters and 100 flyers		130
	ORS souvenirs: 100 plastic teaspoons and cups		25
	100 disaster kits		380
	Polaroid films		100
